# Analysis of risk factors leading to postoperative urethral stricture and bladder neck contracture following transurethral resection of prostate

**DOI:** 10.1590/S1677-5538.IBJU.2014.0500

**Published:** 2016

**Authors:** Huang Tao, Yu Yong Jiang, Qi Jun, Xu Ding, Duan Liu Jian, Ding Jie, Zhu Yu Ping

**Affiliations:** 1 Department of Urology, Anhui Provincial Hospital, Hefei, Anhui, China; 2 Department of Urology, Xin Hua Hospital Affiliated to Shanghai Jiao Tong University School of Medicine, Shanghai, China

**Keywords:** Prostatic Hyperplasia, Transurethral Resection of Prostate, Postoperative Complications, Dysuria, Risk Factors

## Abstract

**Purpose:**

To determine risk factors of postoperative urethral stricture (US) and vesical neck contracture (BNC) after transurethral resection of prostate (TURP) from perioperative parameters.

**Materials and Methods:**

373 patients underwent TURP in a Chinese center for lower urinary tract symptoms suggestive of benign prostatic obstruction (LUTS/BPO), with their perioperative and follow-up clinical data being collected. Univariate analyses were used to determine variables which had correlation with the incidence of US and BNC before logistic regression being applied to find out independent risk factors.

**Results:**

The median follow-up was 29.3 months with the incidence of US and BNC being 7.8% and 5.4% respectively. Resection speed, reduction in hemoglobin (ΔHb) and hematocrit (ΔHCT) levels, incidence of urethral mucosa rupture, re-catheterization and continuous infection had significant correlation with US, while PSA level, storage score, total prostate volume (TPV), transitional zone volume (TZV), transitional zone index (TZI), resection time and resected gland weight had significant correlation with BNC. Lower resection speed (OR=0.48), urethral mucosa rupture (OR=2.44) and continuous infection (OR=1.49) as well as higher storage score (OR=2.51) and lower TPV (OR=0.15) were found to be the independent risk factors of US and BNC respectively.

**Conclusions:**

Lower resection speed, intraoperative urethral mucosa rupture and postoperative continuous infection were associated with a higher risk of US while severer storage phase symptom and smaller prostate size were associated with a higher risk of BNC after TURP.

## INTRODUCTION

Been widely used since the 1970s, transurethral resection of prostate (TURP) has been the most popular treatment of lower urinary tract symptoms suggestive of benign prostatic obstruction (LUTS/BPO). In recent years, new endouro-logic procedures including photoselective vaporization of the prostate (PVP), holmium laser enucleation of the prostate (HoLEP), PVP with the green light laser, transurethral microwave thermotherapy (TUMT), transurethral needle ablation (TUNA), etc. have been applied and found to be safe and effective from relevant randomized controlled trials (RCT) (1–6). However, TURP is still the major surgical method in developing countries including China for its lower cost and favorable outcome.

Comparing with initial application, the mortality of TURP has declined remarkably. Also, incidence of complications has decreased on account of developments in both surgical experience and endoscopic devices (7). Postoperative dysuria is often caused by urethral stricture (US) and bladder neck contracture (BNC), the incidences of which were reported from 0.3 to 9.2% and 2.2 to 9.8% respectively, in contemporary literature (8–10). Dysuria can severely impair surgical efficacy and patient's life quality, sometimes leads to re-operation and even fatal complications. To current knowledge, US is induce by excessive scar formation which is mainly the result of intraoperative urethral mucosa injury and continuous inflammation while the mechanism of BNC formation has not yet been completely understood (7). Many factors have been verified to correlate with high risks of US or BNC including unmanaged preoperative infection, unsuitable diameters of resectoscope, long resection duration time and postoperative catheterization, etc. In present research, we aimed to investigate the influence of specific perioperative clinical parameters on the occurrences of US and BNC after TURP, further disclosing available risk factors and assessing their force of inducing US and BNC.

## MATERIALS AND METHODS

### Preoperative data collection

This was a prospective study approved by the ethical committee of Xin Hua Hospital in April 2007. Patients with LUTS/BPO who have entered the urology department of Xin Hua Hospital for surgical therapy were recruited initially from July 2007 to June 2012.

The enrolled subjects have had a standard medication of α-adrenergic blockers (terazosin, doxazosin or tamsulosin) combined with 5α-reductase inhibitors (finasteride or dutasteride) for at least 6 months before surgical therapeutic decision being made based on both clinical assessment and the patient's desire. Either obvious LUTS or recurrent complications including hematuria, infection, bladder calculus and urinary retention, etc. were found in all candidates. Exclusion criteria in preoperative phase included: (1) maximum flow rate (Q_max_) more than 20mL/s; (2) detrusor underactivity (DUA) defined as detrusor pressure at Q_max_ (PdetQ_max_) of <40cmH2O with Q_max_ of <15mL/s (11) secondary to neurogenic bladder dysfunction or other diseases; (3) history of prostatic and/or urethral surgery; (4) previous diagnosis or intraoperative detection of US or BNC; (5) prostatic or bladder malignancy. Eligible candidates were invited to join the research before signing informed consent. After being inquired with a detailed clinical history, LUTS/BPO related subjective symptom were measured by the International Prostate Symptoms Score (IPSS) system consisting of storage and voiding phase score and the quality-of-life (QoL) questionnaires. Objective tests were carried out routinely, including complete blood count, urea, creatinine, electrolytes, bleeding and clotting tests, prostate specific antigen (PSA) and urine analysis. Total prostate volume (TPV), transitional zone volume (TZV) and intravesical prostatic protrusion (IPP) were measured by transrectal ultrasound scan (TRUS, Sequoia 512; Siemens, Cologne, Germany) before transitional zone index (TZI) being calculated as TZV/TPV. Q_max_, average flow rate (Qave), postvoid residual urine volume (PVR) and PdetQ_max_ were measured by urodynamics including free flowmetry, water filling cystometry and pressure flow study relying on a multichannel system (UDS64-III; Laborie, Quebec, Canada). Bladder outlet obstruction index (BOOI, by “PdetQ_max_-2Q_max_”) and the bladder contractility index (by “PdetQ_max_+5Q_max_”) were calculated by equations from the ICS (12).

### Characteristics of surgery

Standard procedure with technique of complete adenoma resection was given by a single surgical team including 3 doctors with over 5 year's TURP operation experience after formal training. Surgery were performed with the use of a 25.6 F continuous irrigation resectoscope (USA Elite System; GYRUS ACMI, Southborough, MA, USA) equipped with standard tungsten wire loop and monopolar energy (Valleylab Force FX, Boulder, CO, USA) settings for cutting and coagulation at 180W and 70W respectively. 5% glucose solution was used as flushing fluid, routine blood glucose monitoring and advanced circulatory loading measurement were applied during the surgery. 22F urethral triple-lumen Foley catheter was placed and continuous irrigation using sterile saline was applied if apparent hematuria occurred postoperatively. Catheter was conventionally removed 72 hours postoperatively after urine had been clear. Antibiotic prophylaxis was applied pre-and 12 hours postoperatively using second generation cephalosporin. Patients were prescribed oral third generation quinolones for two weeks postoperatively.

Resected tissue was squeezed and weighed in operation room immediately after completion of surgery. The real weight of resected gland was calculated as “resected tissue weight×1.2”, due to dehydration by electroresection (13). Resection time and other details of surgery were also recorded. Blood loss was estimated by the reduction in hemoglobin (ΔHb) and hematocrit (ΔHCT) levels before and 24 hours after surgery (14). Resection speed was calculated as tissue weight being divided by resection time. The incidence of intraoperative transfusion, capsule perforation and transurethral resection syndrome (TURS) were recorded.

### Postoperative and follow-up characteristics

Duration of catheterization in hospital, incidence of re-catheterization (caused by serious hemorrhage or retention) and continuous infection (defined as positive urine test persisting over 6 weeks after removal of catheter) were recorded. Patients were asked to enter regular follow-up 3, 6, 12, 18, 24 and 36 months after surgery if no emergent complications such as haematuria, acute retention, symptomatic infection or incontinence, etc. has happened. During follow-up, patients were re-examined with assessment of IPSS, QoL, urine test and free flowmetry. US and BNC were highly suspected if patients complained of aggravating dysuria or Q_max_ less than 10mL/s. Definite diagnosis was made by urethroscopy and urethrography.

### Statistical methods

Perioperative and follow-up data of all subjects are listed in Tables-1 and 2. Patients were grouped according to the occurrence of US and BNC. Kolmogorov–Smirnov goodness-of-fit test was used to determine whether the distribution of a certain variable was normal. Because the number of enrolled variables is large and many variables have mutual correlation between each other, Student's t-test, Mann-Whitney U-test or chi-square test were firstly applied to select continuous or categorical variables significantly affecting the susceptibility to US or BNC (Table-3). The threshold of P value was set to 0.1 in order to avoid improper omission. Then, logistic regression analysis using stepwise forward method was carried out to filter variables from univariate analyses; likelihood ratio tests with a significance level of 0.05 were used to include or remove any of the factors at each step (Table-4). Finally, cut-off value of continuous variables selected from logistic regression was calculated by receiver operator characteristic (ROC) curve (Table-4). All calculation was processed using routines of the IBM SPSS ver. 19.0 (IBM Co., New York, NY, USA).

**Table 1:** Baseline and follow-up data of 373 patients.

		Follow-up			
Variables	Baseline	12~23 months (N=109)	24~35 months (N=138)	36 months (N=126)	P-value***
Age (year)	64.1±6.4				
Serum total PSA (ng/mL)	5.5±2.1				
IPSS	16.6±5.5	5.0±2.9	5.7±2.5	6.3±2.4	0.001*
Storage Score	7.6±2.7	4.5±1.6	4.9±2.2	5.1±2.9	0.024*
Voiding Score	7.2±2.1	1.3±0.8	1.2±0.6	1.7±0.9	0.011*
QoL index	5 (3, 6)	1 (0, 4)	1.5 (0, 4)	1 (0, 5)	0.027**
Ultrasonography					
TPV (mL)	76.5±20.9				
TZV (mL)	42.0±8.3				
TZI	0.53±0.12				
IPP (mm)	16.2±5.7				
Urodynamics					
Q_max_ (mL/s)	6.1±2.1	17.9±5.8	16.6±7.0	16.2±6.1	0.006*
Qave (mL/s)	3.6±1.1				
PdetQ_max_	77.9±16.6				
PVR (mL)	69.7±25.4				
BOOI	91.6±30.9				
BCI	118.3±14.3				

Values are presented as mean±SD or median (range).

**PSA** = total prostate-specific antigen; **IPSS** = International Prostate Symptom Score; **QoL** = quality of life; **TPV** = total prostate volume; **TZV** = transitional zone volume; **TZI** = transitional zone index; **IPP** = intravesical prostatic protrusion; **Q**_max_ = maximum urinary flow; Qave = average urinary flow; **PdetQ**_max_ = pressure of detrusor at Q_max_; **PVR** = postvoiding residual; **BOOI** = bladder outlet obstruction index; **BCI** = bladder contractility index.

* Student's t-test;

** Mann-Whitney U-test;

*** 36 months' follow-up vs baseline.

**Table 2:** Peri-operative data of 373 patients.

Variables	
Resection time (min)	56.4±10.2
Resected gland weight (g)	47.3±8.7
Resection speed (g/min)	0.82±0.11
ΔHb (g/dL)	-(1.6±0.24)
ΔHCT (%)	-(6.0±2.3)
Incidence of urethral mucosa rupture	48 (12.9)
Incidence of capsule perforation	36 (9.7)
Incidence of TURS	13 (3.5)
Incidence of transfusion	23 (6.2)
Duration of catheterization in hospital (d)	3 (3, 12)
Incidence of re-catheterization	23 (6.2)
Incidence of countinous infection	31 (8.3)

Values are presented as mean±SD, median (range) or number (%).

**Hb** = hemoglobin; **HCT** = hematocrit; **TURS** = transurethral resection syndrome.

**Table 3:** Data comparison of patients divided by the occurrence of us and BNC during follow-up.

Parameters	BNC		
	With	Without	P-value
N (%)	20 (5.4)	353 (94.6)	
Serum total PSA (ng/mL)	5.8±2.0	3.6±1.3	0.049*
IPSS	18.6±4.1	17.2±3.5	0.340*
Storage Score	10.3±1.9	6.5±1.4	0.042*
TPV (mL)	36.8±14.5	68.3±17.1	0.038*
TZV (mL)	37.2±9.0	46.1±14.6	0.047*
TZI	0.48±0.11	0.59±0.15	0.063*
Resection time (min)	48.7±10.1	61.6±11.7	0.051*
Resected gland weight (g)	39.2±6.8	48.3±7.7	0.030*
	US		
	With	Without	P-value
N (%)	29 (7.8)	344 (92.2)	
Resection speed (g/min)	0.75±0.13	0.84±0.19	0.041*
ΔHb (g/dL)	1.59±0.58	1.53±0.46	0.061*
ΔHCT (%)	6.3±1.9	5.9±1.4	0.078*
Incidence of urethral mucosa rupture (n)	7	41	0.059**
Incidence of re-catheterization (n)	4	19	0.092**
Incidence of countinous infection (n)	10	21	0.000**

Values are presented as mean±SD or number.

**US** = urethral stricture; **BNC** = bladder neck contracture; **PSA** = total prostate-specific antigen; **IPSS** = International Prostate Symptom Score; **TPV** = total prostate volume; **TZV** = transitional zone volume; **TZI** = transitional zone index; **Hb** = hemoglobin; **HCT** = hematocrit.

* Student's t-test;

** chi-square test.

**Table 4:** Multiple stepwise forward logistic regression analysis of factors influencing the incidence of postoperative US and BNC.

Parameters	Incidence of US		
	OR (95% CI)	P-value	Cut-off value
Resection speed (g/min)	0.48 (0.30-0.69)	0.022*	0.78
ΔHb	2.08 (0.65-3.41)	0.495	
ΔHCT	1.79 (0.62-1.98)	0.102	
Intraoperative mucosa rupture	2.44 (1.63-3.97)	0.023*	
Postoperative re-catheterization	2.67 (0.58-2.80)	0.761	
Postoperative countinous infection	1.49 (1.21-1.74)	0.028*	
		Incidence of BNC	
Serum total PSA	3.22 (0.38-6.51)	0.291	
Storage score	2.51 (1.37-3.66)	0.028*	8.3
TPV (mL)	0.15 (0.06-0.82)	0.035*	46.2
TZV	0.54 (0.33-1.76)	0.132	
TZI	0.53 (0.30-1.68)	0.157	
Resection time	0.76 (0.31-3.79)	0.145	
Resected gland weight	0.55 (0.24-3.63)	0.304	

**US** = urethral stricture; **BNC** = bladder neck contracture; **Hb** = hemoglobin; **HCT** = hematocrit; **PSA** = total prostate-specific antigen; **TPV** = total prostate volume; **TZV** = transitional zone volume; **TZI** = transitional zone index.

* P<0.05.

## RESULTS

Primarily 407 patients have been enrolled. Nine and 7 patients were excluded because of post-surgery pathologically confirmed prostate and bladder cancer respectively, remaining 391 subjects with no intraoperative mortality. During follow-up period, 2 patients died due to reasons unrelated with prior surgery, 10 were lost to follow-up and 6 were unwilling or unable to participate; 373 remained available for long-term outcome review and contributing to the final analyses. All subjects with mean age of 64.1 years old have undergone TURP, and no seriously complication was observed. At the end of data collection, mean follow-up period was 29.3 months, consisting of 109, 138 and 126 patients with follow-up period over 12, 24 and 36 months respectively. US and BNC were diagnosed in 29 and 20 patients (including 5 patients having both complications) with mean diagnostic time of 2.2 and 3.7 months postoperatively.

**Figure 1:**
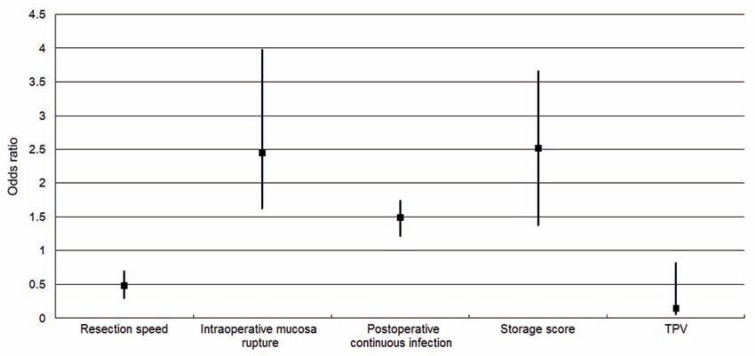
Odds ratio value of independent risk/protective factors about postoperative urethral stricture and bladder neck contracture.

**Figure 2:**
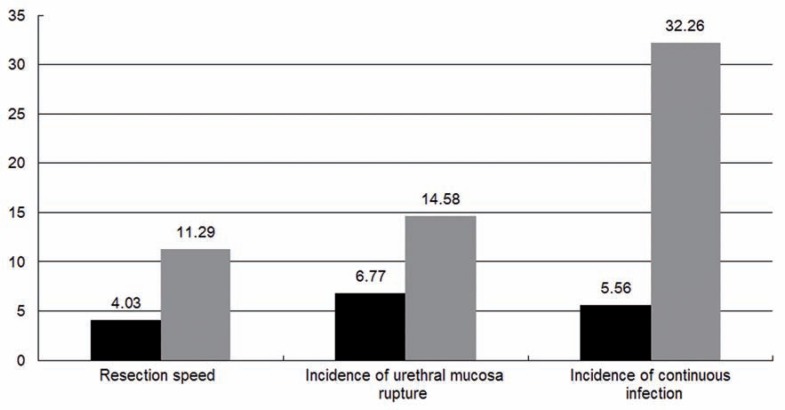
The incidence (%) of urethral stricture between patients with independent risk factors or not.

**Figure 3:**
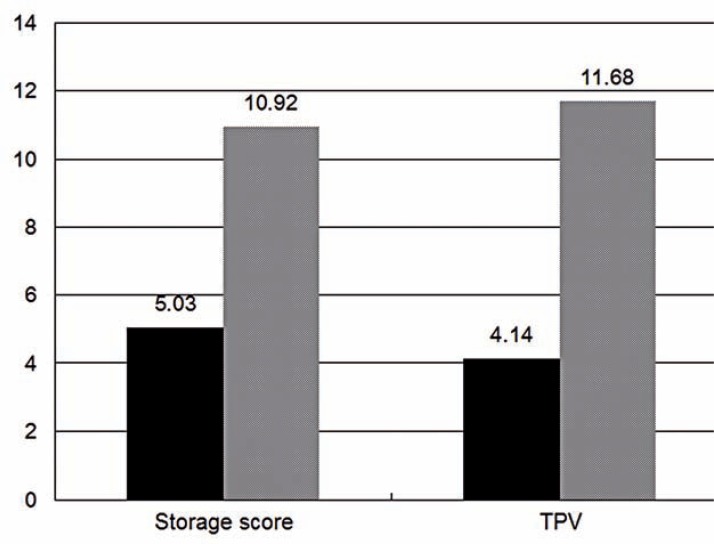
The incidence (%) of bladder neck contracture between patients with independent risk factors or not.

Patients were stratified into different subgroups according to the occurrence of US or BNC. No preoperative factors were found to be significantly different between patients with US or not from univariate analysis (P>0.1), while some operative factors were found to have significant correlation with the occurrence of US and BNC (P<0.1, Table-3). After the analysis of multivariate variables, urethral mucosa rupture and continuous infection were identified as final risk factors of US while higher storage score and lower TPV were found to correlate with higher incidence of BNC (Figures-1, 2 and 3).

## DISCUSSION

LUTS/BPO is a common condition in aging male population. TURP has dominated surgical treatment of LUTS/BPO for over 70 years (15). With the innovation of medicines including α-adrenergic blockers, 5α-reductase inhibitors, plant medicines, etc. medication has become the first-line therapy of LUTS/BPO gradually, especially in most developed countries (16). However, TURP still plays an important role in LUTS/BPO patients either being refractory to medication or having severe complications in some developing countries.

High mortality of TURP was observed during the period of its initial use, which has been reported as 2.5% by Holtgreve et al. among 2.015 cases in 1962 (17). Fortunately, this value has decreased substantially during the past few decades to 0.25% in contemporary series (18), mainly attributable to the improvements of anesthesia and surgical procedure (19). Along with the development of safety, surgical efficacy and long-term outcome have also improved remarkably due to numerous technical progression including video––assisted system, continuous-flow instruments, special loop designs, modifications of high-frequency generators and appropriate perioperative medical treatment (20), thus making TURP one of the most common surgical methods worldwide and the “gold standard” in treating LUTS/BPO, with>90% of the patients reporting normal or improved voiding after 10 year follow-up period (5, 21). Although certain minimally invasive and endoscopic therapies including HoLEP, PVP, TUMT and TUNA have shown favorable short/long-term results, the “golden standard” status of TURP has not been threatened without cohort studies of large samples (4, 22–25).

As mentioned before, significant technical improvements of TURP have made a major impact on the incidence of associated morbidity. Therefore, the risk of intra/postoperative complication of contemporary TURP is lower than that reported previously (20). In addition to technical complications such as bleeding, capsule perforation, TURS, clot retention, urinary tract infection, hydronephrosis, urosepsis and incontinence, associated morbidity consisting of cardiac arrhythmia, myocardial infarction, pulmonary embolism, pneumonitis, chronic obstructive pulmonary disease (COPD) and deep vein thrombosis have been documented in 0.5% to 11% of patients postoperatively (26–29).

Another important postoperative long––term complication is failure to void, often caused by US or BNC. In former literature, US rate ranges from 2.2 to 9.8% while BNC from 0.3 to 9.2% (5). Meatal strictures usually occur because of an inappropriate relationship between the size of the instrument and the diameter of the urethral meatus, while bulbar strictures occur because insufficient isolation by the lubricant causes the monopolar current to leak. To avoid this complication, lubricant should be applied sufficiently in the urethra and along the outer sheath of resectoscope and reapplied in cases of longer resection time. High cutting current should be avoided and an internal urethrotomy must be performed before TURP if there are pre-existing meatal or urethral strictures (30).

In this cohort, 29 cases developed postoperative stricture. Urethral mucosa rupture has been found as independent risk factor of US, which is in accord with the fact that once urethral mucosal integrity is lost, there would be urine leakage underneath the epithelium and subsequently inflammation and scar formation (31, 32). Lower re-section speed was another risk factor, perhaps due to its correlation with unfavorable surgical process including hemorrhage, poor vision, prolonged operative time, more fluid leakage/absorption and urethral mucosal impairment, all of which are potential reasons of US. Because of a wound, former prostatic infection and continuous catheterization, there will always be a temporary local infection post-operation and after catheter removal. However, slowly wound healing, long-time clot retention as well as long-period catheterization etc. will probably lead to continuous infection and result in US. In this research, continuous infection was found as the last risk factor of US.

BNC usually happens after smaller glands are resected. Therefore, the indication for TURP in cases of smaller glands should be taken very seriously. A prophylactic bladder neck incision at the end of the procedure may reduce the incidence. Treatment includes electrical, or preferably, laser incision of the bladder neck (33). Except for lower TPV, we have found higher storage score another risk factor of BNC in this cohort, perhaps due to the negative correlation between storage score and BOOI or TPV (data not shown).

In order to avoid potential bias caused by different surgeons, we have classified all patients into subgroups according to their operator. Not only perioperative parameters but also the incidence of US and BNC were found to have no difference between 3 subgroups (data not shown). However, this research still has limitations, especially on the small quantity of subjects and incidence of US and BNC. Further studies with larger samples and higher power are required to verify the result of this research.

## CONCLUSIONS

Lower resection speed, intraoperative urethral mucosa rupture and postoperative continuous infection were associated with a higher risk of US while severer storage phase symptom and smaller prostate size were associated with a higher risk of BNC.
